# Botulinum Toxin A Facilitated Laparoscopic Repair of Complex Ventral Hernia

**DOI:** 10.3389/fsurg.2021.803023

**Published:** 2022-01-10

**Authors:** Fu-Xin Tang, Ning Ma, Enmin Huang, Tao Ma, Chuang-Xiong Liu, Shuang Chen, Zhen Zong, Tai-Cheng Zhou

**Affiliations:** ^1^Guangdong Provincial Key Laboratory of Colorectal and Pelvic Floor Diseases Supported by National Key Clinical Discipline, Department of Colorectal Surgery, The Sixth Affiliated Hospital, Guangdong Institute of Gastroenterology, Sun Yat-sen University, Guangzhou, China; ^2^Guangdong Provincial Key Laboratory of Colorectal and Pelvic Floor Diseases Supported by National Key Clinical Discipline, Department of Gastrointestinal Surgery and Hernia Center, The Sixth Affiliated Hospital, Guangdong Institute of Gastroenterology, Sun Yat-sen University, Guangzhou, China; ^3^Department of Gastrointestinal Surgery, The Second Affiliated Hospital of Nanchang University, Nanchang, China

**Keywords:** complex ventral hernia, incisional hernia, botulinum toxin A, preoperative progressive pneumoperitoneum, laparoscopic repair

## Abstract

**Background:** Complex ventral hernia repair can be challenging despite the recent advances in surgical techniques. Here, we aimed to examine the effectiveness of preoperative combined use of botulinum toxin A (BTA) and preoperative progressive pneumoperitoneum (PPP) for surgical preparation of patients with complex ventral hernia.

**Methods:** In this prospective, observational study, we included 22 patients with complex ventral hernia between January 2018 and May 2021. All patients were treated with BTA injections into the lateral abdominal muscles and PPP before hernia repair. The lengths of abdominal wall muscles, the volumes of the incisional hernia (VIH), the volumes of the abdominal cavity (VAC), and the VIH/VAC ratio were measured before and after BTA and PPP using abdominal CT scan. All Hernias were repaired using laparoscopic intra-peritoneal onlay mesh (IPOM) or laparoscopic-open-laparoscopic (LOL) techniques.

**Results:** Imaging showed a significant increase in the mean lateral abdominal muscle length from 13.1 to 17.2 cm/side (*p* < 0.01). Before and after BTA and PPP, the mean VIH was 894 cc and 1209 cc (*P* < 0.01), and the mean VAC was 6,692 cc and 9,183 cc (*P* < 0.01). The VAC increased by 2,491 cc (P < 0.01) and was greater than the mean VIH before PPP. An average reduction of 0.9% of the VIH/VAC ratio after BTA and PPP was obtained (*p* > 0.05). All hernias were surgically reduced with mesh, hernia recurrence occurred in only two patients.

**Conclusions:** The preoperative combined use of PPP and BTA increased the abdominal volume, lengthened the laterally retracted abdominal muscles, and facilitated laparoscopic closure of large complex ventral hernia.

## Introduction

An incisional hernia is a common complication following abdominal surgery, with a 5-year incidence of 33.0% in patients undergoing open rectal cancer resection and 13.0% in laparoscopic resection (1). Although a surgical mesh and component separation are widely used, high rates of hernia recurrence and morbidity remain considerable challenges. Recent studies have reported that the recurrence rates of incisional hernias following open repair and laparoscopic repair are 9–12.3% and 7–10.6%, respectively (2, 3). Some hernias gradually develop into large incisional hernias (LIH) or complex ventral hernias, making repair more difficult (4). Chronic contractions of the abdominal muscles reduce the volume of the abdominal cavity and increase the difficulty of performing fascial closure, resulting in an increased risk of postoperative morbidities such as insufficient respiration and abdominal compartment syndrome (ACS) or failed hernia repair (5). In these patients, adequate preparation prior to surgery is vital.

Preoperative progressive pneumoperitoneum (PPP) first described by Goñi Moreno in 1947, is mainly used in preoperative preparation of large incisional hernias (6). PPP can increase the volume of the abdominal cavity, facilitate the reintroduction of the hernia contents into the abdominal cavity, and improve respiratory adaptation; thus, the possibility of postoperative ACS is substantially reduced in patients with large incisional hernias (5, 7).

In recent years, a new technique involving botulinum toxin A (BTA) has been described for preoperative abdominal muscle relaxation. BTA is a neurotoxin that blocks acetylcholine receptors at the neuromuscular junction; when injected preoperatively into abdominal muscles, it causes flaccid paralysis and lengthening of the lateral abdominal muscles, thereby facilitating surgical closure and repair (8–13).

In the present prospective study, we aimed to examine the effectiveness of preoperative combined use of BTA injection and PPP in the management of complex ventral hernia.

## Patients and Methods

### Patients

In this single-center, prospective, observational study, we included a total of 22 patients who were administered BTA injections into their lateral abdominal muscles and PPP prior to elective ventral hernia repair between January 2018 and May 2021. All patients presented with complex ventral hernias according to a consensus paper on the definition of complex abdominal wall hernias (4), and involved a minimum linear defect (or sum of multiple defects) of 6 cm or greater, as measured on abdominal CT imaging. Informed consent was obtained from each patient for inclusion in the study.

### Abdominal Wall Imaging

**Figure 1:**
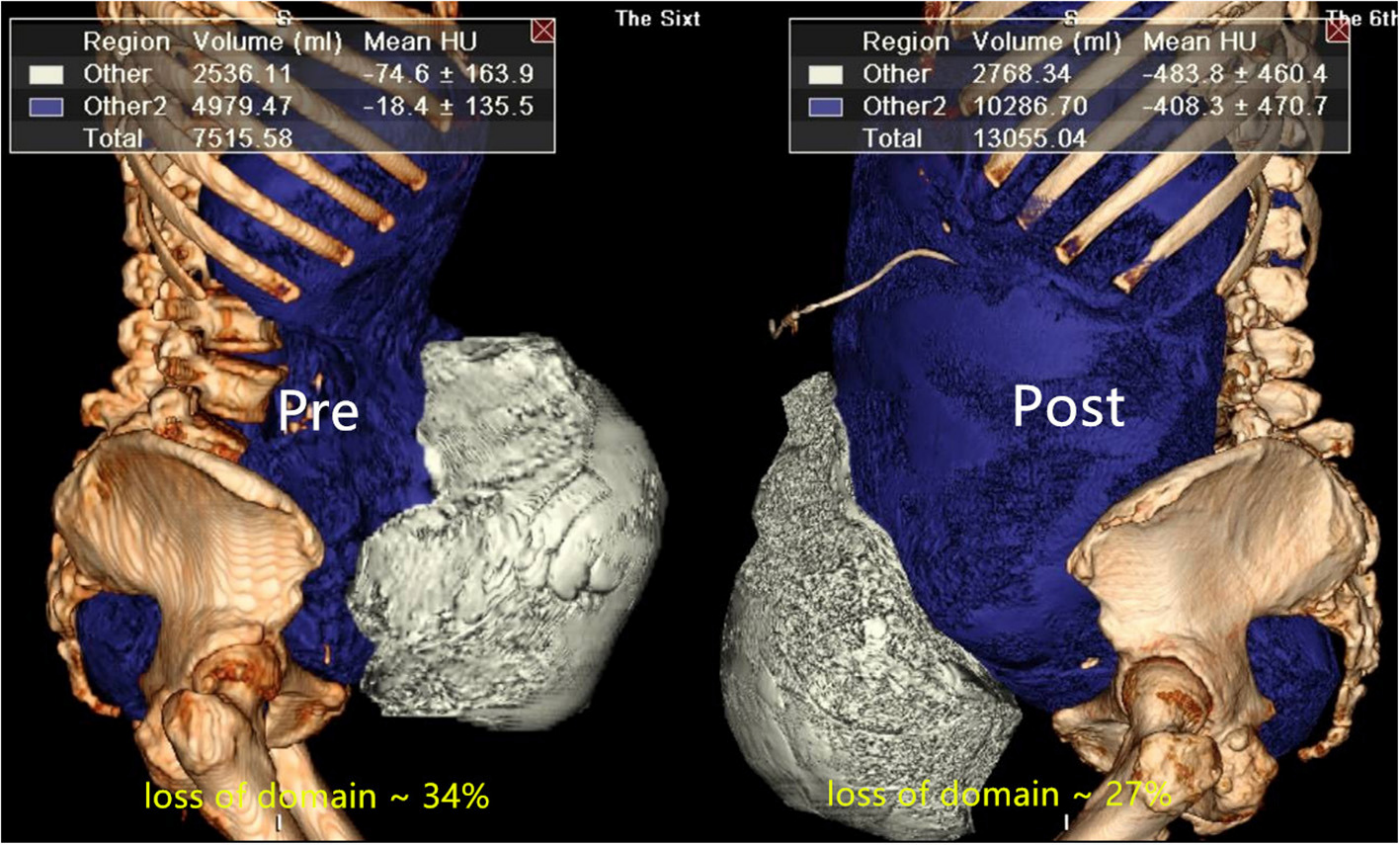
The abdominal volumes (VAC, VIH) were calculated from 64-slice multi-detector CT scan using specific software before and after preoperative techniques.

Before and after BTA and PPP, the changes in lateral abdominal muscle length and thickness, as well as the volume of the incisional hernia (VIH) and volume of the abdominal cavity (VAC) were calculated from non-contrast 64-slice multi-detector CT scan using a specific software (Figure 1). A radiologist, who specialized in abdominal wall scanning, calculated the diameters and volumes according to the abdominal CT findings, based on a modified index of Tanaka (14). Axial muscle length was measured along the deep surface of the abdominal muscle complex from the lateral edge of the quadratus lumborum to the medial edge of rectus abdominis muscle on each side at the same spinal level. Further, muscle thickness was measured along the mid-axillary line from the deep surface of the transversus muscle to the superficial surface of the external oblique muscle. If the VIH/VAC ratio was higher than or equal to 20%, regardless of the size of the hernia defect, he was included in the protocol of preoperative techniques.

### BTA and PPP Management

**Figure 2:**
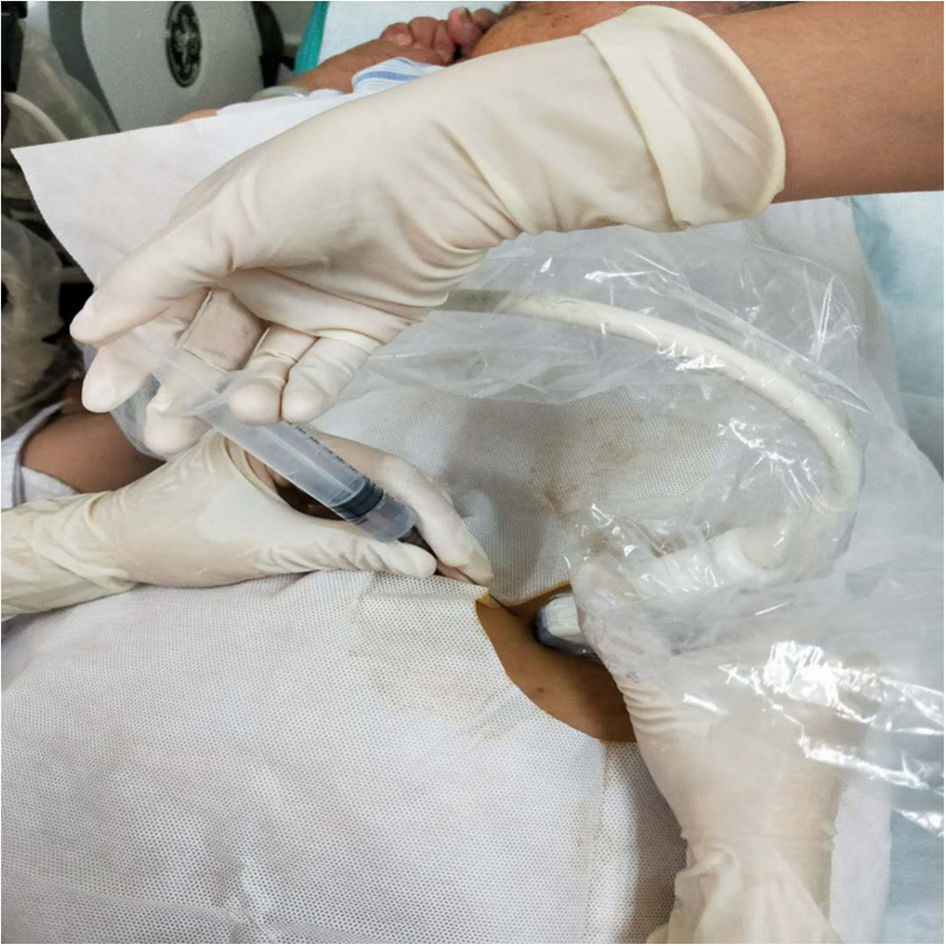
Ultrasound-guided injection of botulinum toxin A into the lateral abdominal muscles. EOM, external oblique muscle; IOM, internal oblique muscle; TM, transverse muscle.

Patients received BTA injections and PPP as an inpatient procedure 2–3 weeks prior to the planned date of surgery. BTA injection and PPP catheter were performed simultaneously in most patients. Most patients received a total dose of 100 or 150 units of BTA (Botulinum Toxin Type A for injection, BOTOX^®^, Allergan, Ireland), which was diluted to 2 units/mL with 0.9% saline and divided into six equal amounts (Relation Botox to Dysport is 1:2.54). Most patients received injections bilaterally. In the early stages of this research, two patients received a total dose of 300 units BTA (Hengli^®^, Lanzhou Biological Products Co. LTD, China). Using real-time ultrasound guidance, three sites were identified on each side of the abdomen along the anterior axillary line equidistant from the costal margin at the level of the ninth rib and a point anterior to the anterior superior iliac spine, as previously identified (15). Then, 12 mL of the diluted BTA was injected at the three sites of the three lateral oblique muscles (transversus abdominis, internal oblique, and external oblique) at each of the six points (25 units/12 mL at each injection site) (Figure 2), and the patient was returned to a supine position.

Then, a 14Fr deep venous catheter was introduced as an intraperitoneal catheter. Under ultrasound guidance, it was preferably placed in the upper left abdomen distant from the previous incision sites. PPP was performed 2–3 weeks prior to surgery. We initially insufflated 200 mL of air and then performed X-ray with the patient in the standing position to check whether PPP was appropriately performed. Approximately, the total volume of insufflation was four times the volume (cc of hernia sac (VIH) in CT findings. Subsequently, according to individual tolerance, we insufflated 400 mL of air daily using a 50 mL syringe. The patients were discharged for the next 24–48 h. During the period of PPP administration, an abdominal support belt was used to reduce air accumulation in the hernia sac, and were encouraged to improve their respiratory function and to walk for at least 2 h a day. We tried our best to reduce the loss of domain ratio, so that the increase in abdominal volume was greater than the mean VIH before PPP. The duration of PPP administration was 2–3 weeks until the surgery. After the insufflation, the length, thickness and the abdominal volumes were evaluated again by CT scan the day before the patient was programmed for surgery.

PPP administration was temporarily terminated when the following instances occurred: (1) intolerable subjective symptoms such as abdominal pain, abdominal distension, and shoulder pain; (2) dyspnea, hypoxemia, or CO2 retention; and (3) serious subcutaneous emphysema.

### Surgical Procedure

All patients underwent laparoscopic intra-peritoneal onlay mesh (IPOM) or laparoscopic-open-laparoscopic (LOL) hernia repair. In brief, the patient was placed in the supine position and an indwelling urinary catheter was routinely inserted after intubation. Initial insufflation was undertaken using an intraperitoneal catheter. A routine 12-mm camera port and two 5-mm working ports were inserted as required under direct vision. Then, the hernia contents were reduced into the abdominal cavity. Fascial closure was achieved using non-absorbable sutures in a transverse shoelace pattern along the length of the defect. Intra-peritoneal onlay mesh (IPOM) was preferably performed with non-absorbable tacks (Covidien, USA) in double crown arrangement. After the primary closure, mesh repair was carried out using a composite-polypropylene mesh (Bard Sepramesh™ or Parietex™), a few patients were treated with biological mesh and covering the defect at least 5 cm all around. All surgeries were performed by the same team of doctors.

Postoperatively, all patients were fitted with an abdominal binder for 6 months following repair. Intra-abdominal pressures were monitored by measuring bladder pressure using an indwelling urinary catheter, as dictated by comorbidities. Antibiotic prophylaxis was continued for 48 h or longer if indicated and enoxaparin was routinely given for deep vein thromboembolic prophylaxis continued throughout admission.

### Statistical Analysis

Descriptive statistics, including means and standard deviations, were used for continuous variables. The changes in lateral abdominal muscle length, as well as the VIH and the VAC before and after administration were measured. Each patient served as his own control. Univariate analysis was performed using Student's *t*-test or Wilcoxon's test to explore quantitative variables and chi-square or Fisher's test if data were dichotomous. All analyses were performed using the SPSS version 20.0 software (IBM Corp., Armonk, NY, USA). A *p* < 0.05 was considered statistically significant.

## Results

### Demographic and Characteristics of Patients

A total of 22 patients (12 males and 10 females) received preoperative combined abdominal wall BTA injections and PPP treatment. The details of the 22 patients are listed in Table 1. The mean patient age was 64.9 years (range 44–82 years) and mean BMI was 26.8 kg/m^2^ (range 21.3–37.7 kg/m^2^). Twenty-two patients presented with ventral incisional hernias, with 11 midline defects, and 11 lateral defects. The mean time since occurrence of the incisional hernia was 26.5 months (range 1–216). One patient in our study had one previous failed repair; two patients had two failed repairs. The smallest fascial defect was 11 × 9 cm in size and the largest was 13 × 17 cm.

**Table 1:** Demographics and characteristics of the patients with complex ventral hernia.

Sex	N (%)
Male	12 (54.5)
Female	10 (45.5)
Median previous mesh-repair	0.2 ± 0.6 (0–2)
Transverse defect	
>10 cm	22 (100.0)
First abdominal surgery	
Tumor excision	8 (36.4)
Exploratory Laparotomy	11 (50.0)
Choledocholithotomy	1 (4.5)
Appendicectomy/ Cholecystectomy	2 (9.1)
Evolution time of hernia, months	26.5 ± 50.0 (1–216)
Obesity (BMI > 30)	2 (9.1)
Other comorbidities	
Diabetes	2 (9.1)
Hypertension	5 (22.7)
History of abdominal sepsis	1 (4.5)

Among the patients, two patients had diabetes and five patients had hypertension. Moreover, eight patients had undergone abdominal tumor resection. Five patients developed complications after the first abdominal surgery, three patients had incisional complications, one patient had abdominal cavity infection and one patient had bile leakage.

### Changes in Lateral Abdominal Wall Muscle and Abdominal Volume

**Figure 3:**
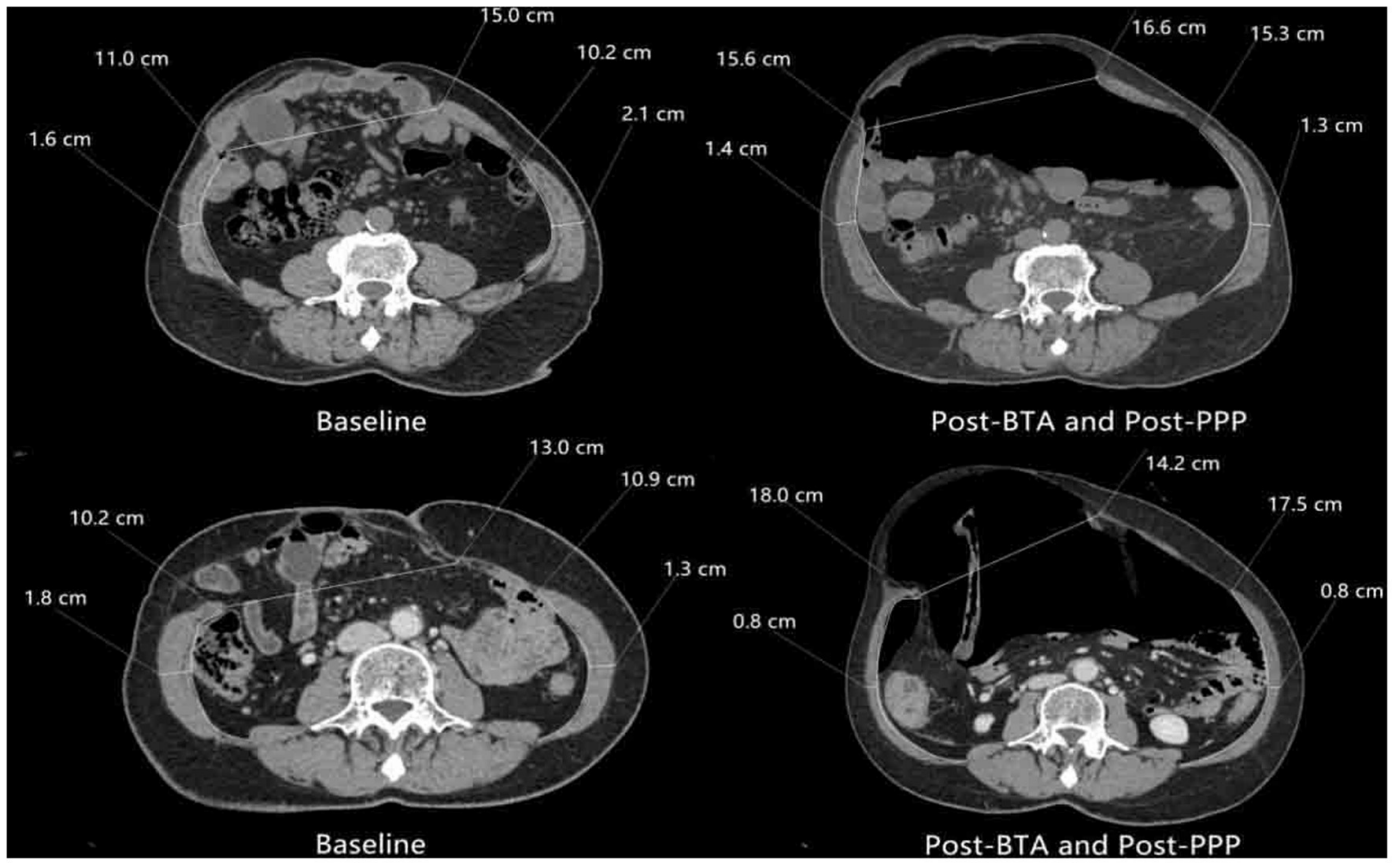
Comparison of preoperative axial computed tomography image before and after preoperative techniques. There is significant elongation and thinning of the lateral abdominal oblique muscles.

On comparing CT images before and after the administration of combined BTA injection and PPP, we noted increased lateral abdominal muscle length and simultaneous decreased muscle thickness after the combination approach (Figure 3). There was a significant increase in the mean abdominal muscle length from 13.1 ± 2.6 cm/side (range 9.1–17.7 cm/side) before the combination approach to 17.2 ± 2.6 cm/side (range 12.3–23.2 cm/side) after the combination approach (*p* < 0.01), indicating a gain in the mean transverse length of the lateral abdominal muscles of 4.1 cm/side (range 1.5–7.2 cm/side) (Table 2). In the patients with midline hernias (*n* = 11), the mean absolute gain in lateral abdominal muscle length prior to surgery was 3.8 cm/side (range 1.5 cm−6.8 cm) (*p* < 0.01).

**Table 2:** Results of all 22 patients undergoing pre-operative BTA injection and PPP treatment.

**Patient**	**Hernia size (width × length, cm)**	**Length (left) (cm)**			**Length (right) (cm)**		
		**Pre-BTA**	**Post-BTA**	**Difference**	**Pre-BTA**	**Post-BTA**	**Difference**
PT-1	14 × 8	18.3	23.5	5.2	16.9	22.8	5.9
PT-2	13 × 15	13.8	15.6	1.8	11.6	16.3	4.7
PT-3	10 × 12	17.4	22.0	4.6	17.9	19.8	1.9
PT-4	12 × 15	11.9	15.3	3.4	12.5	18.1	5.6
PT-5	11 × 10	15.5	18.7	3.2	17.3	20.6	3.3
PT-6	13 × 13	15.5	18.8	3.3	15.7	19.6	3.9
PT-7	13 × 16	10.9	17.5	6.6	10.2	18.0	7.8
PT-8	11 × 16	9.2	14.6	5.4	12.8	15.6	2.8
PT-9	15 × 14	10.2	15.3	5.1	11.0	15.6	4.6
PT-10	12 × 11	11.4	17.8	6.4	13.3	20.5	7.2
PT-11	11 × 16	12.8	16.8	4.0	14.7	17.7	3.0
PT-12	10 × 11	15.7	18.3	2.6	15.3	20.8	5.5
PT-13	15 × 10	13.0	18.8	5.8	8.7	13.1	4.4
PT-14	11 × 13	12.2	17.4	5.2	13.5	17.7	4.2
PT-15	11 × 9	12.1	15.2	3.1	11.2	13.4	2.2
PT-16	11 × 13	11.2	13.1	1.9	8.7	13.6	4.9
PT-17	13 × 17	11.6	15.4	3.8	10.1	16.4	6.3
PT-18	11 × 9	15.1	17.3	2.2	14.4	18.4	4.0
PT-19	11 × 12	9.5	12.8	3.3	8.6	11.8	3.2
PT-20	11 × 10	15.6	20.5	4.9	17.1	19.7	2.6
PT-21	12 × 13	10.8	14.5	3.7	13.8	15.7	1.9
PT-22	10 × 10	14.5	15.3	0.8	14.0	16.1	2.1
Mean				3.9			4.2

*Comparison of lateral abdominal muscle length before and after the preoperative combined use of BTA injection and PPP treatment are show, as measured on CT imaging. All patients except patients 2, 3, and 5 received bilateral BTA injections*.

*PT, patient*.

**Figure 4:**
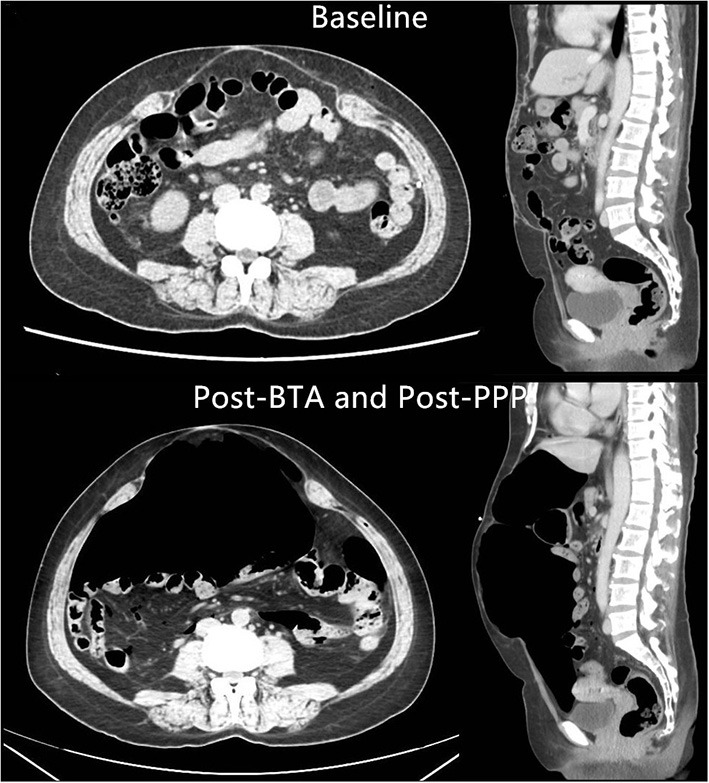
Abdominal CT image comparison of pre-and post-PPP treatments demonstrating spontaneously reinstate the herniated contents (PT-2).

The mean insufflated air volume for PPP was 6827 ± 1574 mL (range 3600–9200 mL) over 18.0 ± 6.8 days (Range 9–43 days). The mean BTA administration time was 16.0 ± 3.9 days (range 9–23 days). A comparison of peritoneal volumes before and after preoperative techniques is shown in Table 3. Before and after PPP, respectively, the VIH was 894 ± 640 cc (216–2,536) and 1,209 ± 941 cc (150–3,271) (*p* < 0.01), the VAC was 6,692 ± 1931 cc (3780–12,748) and 9,183 ± 2119 cc (5,412–15,596) (*p* < 0.01) and the VIH/VAC ratio (loss of domain ratio) was13.5 ± 8.5% (3.0–34.0) and 12.6 ± 8.2% (2.0–27.0) (*p* > 0.05). An average reduction of 0.9 % of the VIH/VAC ratio was observed on CT scan after the combination of BTA and PPP. Furthermore, abdominal CT image comparison of pre-and post-PPP treatments demonstrating spontaneously reinstate the herniated contents (Figure 4).

**Table 3:** Comparison of peritoneal volumes before and after preoperative techniques.

	**Before BTA +PPP (Range)**	**After BTA +PPP (Range)**	***P* value**
VIH (cc)	894 ± 640	1209 ± 941	<0.01
	(216–2,536)	(150–3,271)	
VAC (cc)	6,692 ± 1,931	9183 ± 2,119	<0.01
	(3,780–12,748)	(5,412–15,596)	
VIH/VAC ratio (%)	13.5 ± 8.5	12.6 ± 8.2	>0.05
	(3.0–34.0)	(2.0–27.0)	

*P value derived from Student's t test PPP, progressive preoperative pneumoperitoneum; BTA, botulinum toxin A; VIH, Incisional hernia volume; VAC, abdominal cavity volume*.

There were no complications during the administration of BTA, and the injections were well tolerated. PPP complications included shoulder pain (*n* = 2, 9.1%), subcutaneous emphysema (*n* = 1, 4.5%). There were no other PPP complications such as air embolism, intestinal perforation, catheter-related skin infection, and pneumothorax. Further, no PPP complication required intervention.

### Surgical Outcomes

After the combination approach, all patients had successful laparoscopic repair of ventral hernias. The 30-day mortality rate was zero. The mean operation time was 201.4 ± 50.7 min (range 79–296 min) and mean duration of postoperative hospitalization was 7.5 ± 1.6 days (range 5–11 days). Primary fascial closure was possible in all patients. The postoperative complications inside 30 days included abdominal pain (*n* = 1, 4.5%), dyspnea (*n* = 1, 4.5%). One patient (PT-14) developed mesh infection 4 months after surgery (*n* = 1, 4.5%), and underwent mesh removal and incision and drainage of abdominal abscess. Two patients had incisional hernia recurrence 4 months (PT-20) and 6 months (PT-19) after surgery (*n* = 2, 9.1%), respectively. Laparoscopic incisional hernia repair was performed again after PPP treatment. Notably, no patient experienced ACS, cardiopulmonary failure, pulmonary embolism or skin necrosis. There were no other complications or special conditions to date, with a mean follow-up of 18 months (range 5–32 months).

## Discussion

The repair of complex ventral hernias is challenging, and adequate preoperative preparation is vital to avoid serious complications and to obtain satisfactory results after hernia repair. For this reason, the use of BTA and PPP has emerged as a complementary therapy to facilitate this repair (16).

In 2006, Cakmak et al. (17) first reported the benefits of BTA in rats. They showed that BTA increased abdominal volume and reduced abdominal wall tension, facilitating closure of fascial defects with no respiratory compromise. The effect of BTA is apparent in 2–3 days, with the maximum effect by 2 weeks, and the effect lasts for 6–9 months (18). In 2009, Ibarra-Hurtado et al. (8) first reported the application of BTA in 12 patients with abdominal wall hernias after open abdomen management under electromyographic guidance. Abdominal CT showed that a mean decrease in hernia defect size of 5.25 cm was achieved after 4 weeks of BTA application. All patients had successful repair, and no recurrence was noted for 9 months. In 2014, these authors also reported reduction in abdominal muscle thickness and increase in its length after injection of 250 units of BTA in 17 patients, with similar surgical outcomes (9). Farooque et al. (10) also reported that BTA induced a significant decrease in the thickness and increase in the length of lateral abdominal muscles.

Zielinski et al. (19) reported the results of BTA injection administered to 18 patients with an open abdomen 12–24 h after damage-control laparotomy. A total of 300 units of BTA was injected into the lateral abdominal muscles, and primary fascial closure was achieved in 83% of the patients, with a partial closure rate of 6%. In 2016, Elstner et al. (11) reported a series of 27 patients undergoing complex ventral hernia repair who were injected with 300 units of BTA into the lateral abdominal muscles under ultrasound guidance. The pre-BTA and post-BTA (1–4 weeks) CT images demonstrated a significant increase in lateral abdominal length, with a mean length gain of 4.2 cm/side. All patients had successful hernia repair with no early recurrences. In 2017, these authors also reported an increase in abdominal muscle length in 32 patients with similar characteristics after injection of 300 units of BTA, with a mean length gain of 4.0 cm/side. Fascial closure was achieved in all cases, with no hernia recurrences to date (12).

There are some reports on the use of BTA for postoperative pain management in patients undergoing incisional hernia repair. Smoot et al. (15) reported a patient who underwent incisional hernia repair following the injection of 300 units of BTA. Pain scores decreased from 10/10 to 2/10, and the effect was maintained at the 3-month follow-up. Zendejas et al. (13) reported a series of 22 patients who underwent incisional hernia repair with preoperative the injection of 300 units of BTA. These patients had significantly less postoperative pain and required less opioid analgesia compared with controls (*n* = 66).

In our study, BTA helped achieve primary closure in all patients, and all patients underwent successful intraperitoneal mesh repair. Furthermore, there were no complications during BTA administration, and the injections were well tolerated. Combined with PPP, the approach allowed us to obtain an obvious increase in the abdominal volume in all patients, and this is important for repair in patients with loss of domain hernias.

In 1947, the first study reporting the benefits of PPP and its application for large hernias with loss of domain was published by Moreno (6). Since then, this procedure has been modified and used widely (4, 20). PPP induces progressive distension of the abdominal wall that facilitates reintroduction of hernia contents and improves patient tolerance to increased abdominal pressure (21). It also facilitates tension-free surgical repair and reduces the risk of ACS in patients with hernias involving postoperative loss of domain (22). In addition, Rodriguez-Acevedo et al. (23) demonstrated that PPP facilitates pneumatic adhesiolysis and helps identify safe areas for port placement.

Bueno-Lledo et al. (24) reported the use of the combination of PPP and BTA in the management of LIHs. The study included 45 patients with LIHs and demonstrated a mean reduction in the VIH/VAC ratio of 14% after the combined use of PPP and BTA. Fascial closure was achieved in all patients; however, one patient had ACS (bladder pressure, 31 mmHg) associated with acute respiratory distress syndrome and two had hernia recurrence. Subsequently, in 2018, these authors performed an observational study in 70 patients with a mean loss of domain of 29.8% who received PPP and BTA before surgery and reported a significant decrease in the loss of domain rate (25). Interestingly, two studies reported a slight reduction in the transverse diameter of the hernia defect, without statistical significance (0.8 and 0.9 cm, respectively), although the reduction in the loss of domain rate facilitated the reintroduction of hernia contents into the abdominal cavity, improving respiratory adaptation following hernia repair (24, 25). Rodriguez-Acevedo et al. (23) performed a prospective observational study in 56 patients with complex ventral hernias who received BTA injections. Of these patients, 18 received PPP as an adjunct preoperative procedure to BTA and 38 received only BTA. The authors reported that there was no significant difference in terms of the increase in the mean lateral abdominal length (per side) between the groups. Additionally, primary closure was achieved in all patients, with no case of postoperative ACS.

Therefore, our group confirm a significant increase in abdominal cavity volume with the combination of both techniques, and this is important to the surgical repair. The mean VIH/VAC ratio decreased by 0.9%, the PPP-induced gain in abdominal volume (2491 cc) was greater than the volume of incisional hernia before PPP (894 cc). This facilitates the reintroduction of the herniated volume into the abdominal cavity, improving the respiratory adaptation after the fascial closure. Moreover, imaging demonstrated a significant increase in the mean lateral abdominal muscle length from 13.1 to 17.2 cm/side, with a mean gain of 4.1 cm/side, eliminating the need for the component separation technique during the operation.

However, the indications for the application of BTA or PPP in complex hernia management are not clear. An objective method for calculating the abdominal hernia volume was initially provided by Tanaka et al. (14). They recommend the use of PPP when the VIH/VAC ratio is >25%. Rodriguez-Acevedo et al. (23) reported that the indications for PPP are a fascial defect >15 cm and a loss of domain≥20%. Bueno-Lledo et al. (24, 25) also recommend the use of PPP when the loss of domain is ≥20%. In this study, all patients with loss of domain ratio ≥20% and the transverse defect ≥10 cm. We believe that the main benefits of PPP are to increase the volume of the abdominal cavity, facilitate the reintroduction of the hernia contents into the abdominal cavity, and improve respiratory adaptation, which can help reduce intraoperative complications such as enterotomy or ACS and restrictive respiratory disease following complex hernia repair. Owing to time overlap, the independent benefits of BTA and PPP are difficult to understand. Nevertheless, this study demonstrated that the combination of preoperative BTA and PPP is effective and safe, resulting in flaccid relaxation of the lateral abdominal muscles and a significant increase in abdominal cavity volume. A comparison of CT images demonstrated a significant increase in the mean length of the lateral abdominal muscles (4.8 cm/side) with the combination approach, and this increase has the same effect as that of the component separation technique, without altering the anatomical constitution.

The present study has some limitations. First, the sample size was relatively small (*n* = 22). Second, the follow-up period was limited. Nevertheless, the study demonstrated that the combination of BTA and PPP plays a significant role in preoperative preparation for laparoscopic repair of complex ventral hernias.

## Conclusion

Flaccid relaxation of the lateral abdominal wall with the combined use of BTA and PPP before complex ventral hernia repair is a satisfactory outcome that facilitates laparoscopic closure of large hernia defects. The combination approach causes flaccid paralysis and a significant increase in the length of lateral abdominal muscles, eliminating the need for the component separation technique and allowing fascial closure under minimal abdominal tension. Additionally, the benefits of using BTA is that it will be more conducive to wound healing due to less tension. Moreover, PPP induces an insignificant increase in abdominal cavity volume prior to surgery, which facilitates total reintegration of the bowel into the abdomen.

## Data Availability Statement

The original contributions presented in the study are included in the article/supplementary material, further inquiries can be directed to the corresponding author/s.

## Ethics Statement

The studies involving human participants were reviewed and approved by Ethics Committee of the Sixth Affiliated Hospital of Sun Yat-sen University. The patients/participants provided their written informed consent to participate in this study. Written informed consent was obtained from the individual(s) for the publication of any potentially identifiable images or data included in this article.

## Author Contributions

T-CZ and ZZ: conceptualization and funding acquisition. T-CZ: methodology, visualization, and supervision. F-XT: software. NM and EH: validation. TM: formal analysis. C-XL: investigation. F-XT: resources, data curation, writing—original draft preparation, and writing—review and editing. ZZ: project administration. All authors have read and agreed to the published version of the manuscript.

## Funding

This research was funded by Science and Technology Planning Project of Guangdong Province, Grant Number 2021A1515410004; and Science and Technology Planning Project of Guangzhou City, Grant Number 202201010630010045; and Science and Technology Plan of Jiangxi Provincial Healthcare Commission, Grant Number 202130374, and Science and Technology Plan of Jiangxi Provincial Administration of Traditional Chinese Medicine, Grant Number:2020B0132.

## Conflict of Interest

The authors declare that the research was conducted in the absence of any commercial or financial relationships that could be construed as a potential conflict of interest.

## Publisher's Note

All claims expressed in this article are solely those of the authors and do not necessarily represent those of their affiliated organizations, or those of the publisher, the editors and the reviewers. Any product that may be evaluated in this article, or claim that may be made by its manufacturer, is not guaranteed or endorsed by the publisher.
